# Nutrient responding peptide hormone CCHamide-2 consolidates appetitive memory

**DOI:** 10.3389/fnbeh.2022.986064

**Published:** 2022-10-19

**Authors:** Nobuhiro Yamagata, Yasuhito Imanishi, Hongyang Wu, Shu Kondo, Hiroko Sano, Hiromu Tanimoto

**Affiliations:** ^1^Graduate School of Life Sciences, Tohoku University, Sendai, Japan; ^2^Department of Biological Science and Technology, Faculty of Advanced Engineering, Tokyo University of Science, Tokyo, Japan; ^3^Department of Molecular Genetics, Institute of Life Sciences, Kurume University, Kurume, Japan

**Keywords:** CCHa2, CCHa2-R, memory consolidation, dopamine, *Drosophila*

## Abstract

CCHamide-2 (CCHa2) is a protostome excitatory peptide ortholog known for various arthropod species. In fruit flies, CCHa2 plays a crucial role in the endocrine system, allowing peripheral tissue to communicate with the central nervous system to ensure proper development and the maintenance of energy homeostasis. Since the formation of odor-sugar associative long-term memory (LTM) depends on the nutrient status in an animal, CCHa2 may play an essential role in linking memory and metabolic systems. Here we show that CCHa2 signals are important for consolidating appetitive memory by acting on the rewarding dopamine neurons. Genetic disruption of CCHa2 using mutant strains abolished appetitive LTM but not short-term memory (STM). A post-learning thermal suppression of CCHa2 expressing cells impaired LTM. In contrast, a post-learning thermal activation of CCHa2 cells stabilized STM induced by non-nutritious sugar into LTM. The receptor of CCHa2, CCHa2-R, was expressed in a subset of dopamine neurons that mediate reward for LTM. In accordance, the receptor expression in these dopamine neurons was required for LTM specifically. We thus concluded that CCHa2 conveys a sugar nutrient signal to the dopamine neurons for memory consolidation. Our finding establishes a direct interplay between brain reward and the putative endocrine system for long-term energy homeostasis.

## Introduction

CCHamide (CCHa) is the protostome excitatory peptide ortholog known for various arthropod species, including insects (Roller et al., 2008; Ida et al., 2012), crustaceans (Toullec et al., 2017; Nguyen et al., 2018), myriapods (Christie, 2015), and chelicerates (Veenstra, 2016). It has two conserved cysteines and an amidated C-terminal histidine residue (Hansen et al., 2011; Thiel et al., 2019), forming a cyclic peptide held by an intramolecular disulfide bond. In the insect lineage, the CCHa system is duplicated into two specific peptides with two particular receptors, where each of the peptides activates its receptor paralog (Hansen et al., 2011; Ida et al., 2012). CCHa is enriched in the endocrine cells in the midgut (Roller et al., 2008; Li et al., 2013; Veenstra and Ida, 2014; Hung et al., 2020; Titos and Rogulja, 2020; Zhu et al., 2022) or periphery adipose tissue (Sano et al., 2015), suggestive of its role as a part of the enteroendocrine system. The CCHa system affects various physiological functions and related behaviors, including feeding, diuresis, reproduction, and metabolism (Ida et al., 2012; Li et al., 2013; Veenstra and Ida, 2014; Ren et al., 2015; Sano et al., 2015; Fujiwara et al., 2018; Jayakumar et al., 2018; Capriotti et al., 2019; Jin et al., 2020; Titos and Rogulja, 2020; Shahid et al., 2021; Havula et al., 2022; Zhu et al., 2022).

In fruit flies, *Drosophila melanogaster*, CCHa2 is one of the two CCHa paralogs and is crucial in regulating energy metabolism (Ida et al., 2012; Li et al., 2013; Veenstra and Ida, 2014; Ren et al., 2015; Jayakumar et al., 2018; Jin et al., 2020; Havula et al., 2022). CCHa2 is preferentially expressed not only in the adipose tissue and the midgut (Sano et al., 2015) but also in the brain (Ren et al., 2015). CCHa2 responds to glucose nutrition, but not sucralose (Sano, 2015), by increasing its expression via the polyol pathway that activates the master metabolic regulator Mondo (Sano et al., 2022). CCHa2-R, the receptor of CCHa2, is enriched in the brain. Secreted CCHa2 binds to CCHa2-R in the pars intercerebralis, promoting insulin signaling to control the growth and pupariation of the flies ultimately (Sano et al., 2015; Havula et al., 2022). In agreement with this, the bombesin receptor subtype 3, a mammalian ortholog of the CCHa2-R, also plays a central role in energy and glucose metabolism (Ohki-Hamazaki et al., 1997) via the hypothalamus (Piñol et al., 2018). Interestingly, decades of research critically conjoined the bombesin receptor function in memory consolidation (Flood and Morley, 1988; Shumyatsky et al., 2002; Roesler et al., 2004; Ghanbari et al., 2018; Melzer et al., 2021). Abnormalities in the bombesin receptor pathway in patients with Alzheimer’s disease (Roesler et al., 2007) suggest an as-yet-unsolved cognitive feature of the metabolic neuropeptide family. However, any attributes that signify CCHa2’s function in the cognitive process are so far unclear.

In flies, a single cycle of associative training with odor and sugar drives the formation of appetitive short-term memory (STM) and long-term memory (LTM). The sweetness of the sugar causes labile STM, while the nutrition causes stable LTM (Burke and Waddell, 2011; Fujita and Tanimura, 2011) though a non-nutritious sugar can also induce LTM (McGinnis et al., 2016). LTM formation involves *de novo* protein synthesis that is energetically costly (Mery and Kawecki, 2005; Plaçais and Preat, 2013), urging the memory system to take the metabolic state into account prior to gating the LTM (Musso et al., 2015; Plaçais et al., 2017; Sgammeglia and Sprecher, 2022). In accordance, insulin signaling, a crucial regulator of metabolic homeostasis (Rulifson et al., 2002), is implicated in the formation of LTM both in larvae and adult flies (Chambers et al., 2015; Eschment et al., 2020). Neuropeptide F, another important regulator of feeding (Chung et al., 2017; Tsao et al., 2018; Landayan et al., 2021), food-related memory (Krashes et al., 2009; Rohwedder et al., 2015), and lipid metabolism (Yoshinari et al., 2021), also acts as a disinhibitory gate for LTM consolidation (Feng et al., 2021). Thus, metabolic peptide hormones (Lin et al., 2019; Nässel et al., 2019) appear crucial in linking alimentary and memory consolidation processes for triggering LTM.

In this study, we investigated the role of CCHa2 in odor-sugar associative learning. Disruption of the CCHa2 signaling in a subset of dopamine neurons incapacitated the formation of appetitive LTMs in flies that depends on sugar nutrition. Conversely, thermal activation of CCHa2 turned sweetness-induced labile memory into a long-lasting one. Our findings shed light on the hitherto-unknown function of CCHa2 in the mnemonic process, a gatekeeper for stable LTM via an interplay between brain reward and the endocrine system.

## Results

### Selective long-term memory defect in CCHamide-2 mutant flies

**FIGURE 1:**
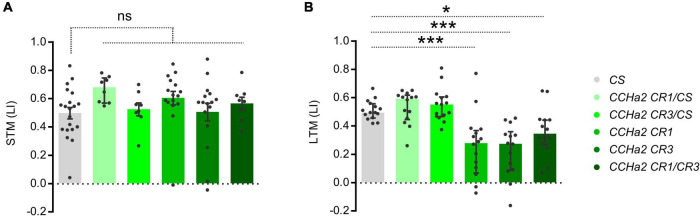
Appetitive LTM defect in CCHa2 mutants. Appetitive STM **(A)** and LTM **(B)** performances of the wild-type strain, heterozygous, homozygous, and trans-heterozygous mutant strains of CCHa2. Median and interquartile range is shown hereafter. ns, not significant, *p < 0.05 (**A:** Dunn’s multiple comparisons test, *N* = 20, 8, 8, 16, 16, 8, **B:** Dunnett’s multiple comparisons test, *N* = 15, 14, 15, 16, 14, 14 and ***p < 0.001).

To examine the role of CCHa2 in appetitive olfactory learning, we first employed CRISPR/Cas9-mediated mutant flies of CCHa2 with null-alleles, which showed characteristic developmental delay (Sano et al., 2015). In the adult, those mutant strains appear normal in locomotion, body weight, and appetitive STM (Figure 1A). However, they exhibited an impaired LTM (Figure 1B) and largely diminished CCHa2 signal in the brain (Supplementary Figure 1), suggesting a role of the CCHa2 signal for the mnemonic rather than sensory or motor processes. The phenotype was recessive, as heterozygous mutant alleles did not affect the memory (Figure 1B).

### CCHamide-2 signaling consolidates appetitive memory

**FIGURE 2:**
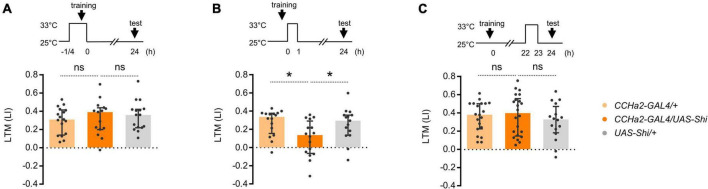
CCHa2 is required for the formation of appetitive LTM. **(A)** Blockade of the CCHa2 neurons in *CCHa2-GAL4/UAS-Shibire[ts1]* flies during learning leaves appetitive LTM intact. ns, not significant (Sidak’s multiple comparisons test, *N* = 17, 15, 16). **(B)** Blockade of the CCHa2 neurons in *CCHa2-GAL4/UAS-Shibire[ts1]* flies for an hour after training impaired appetitive LTM. *p < 0.05 (Sidak’s multiple comparisons test, *N* = 16, 16, 15). **(C)** Blockade of the CCHa2 neurons in *CCHa2-GAL4/UAS-Shibire[ts1]* flies for an hour 22 h after training leaves appetitive LTM intact (Dunn’s multiple comparisons test, *N* = 20, 22, 16).

To understand how CCHa2 regulates LTM, we examined the temporal requirement of CCHa2 neurotransmission during learning and consolidation. To genetically manipulate the activity of CCHa2 expressing neurons, we employed the T2A-GAL4 knock-in strain in the *CCHa2* locus, *CCHa2-T2A-GAL4* (Kondo et al., 2020). We first examined whether CCHa2 mediates the reinforcement signal of sugar for triggering LTM. However, transient thermal blockade of CCHa2 expressing cells during conditioning barely affected the LTM (Figure 2A). We then tested the requirement of CCHa2 signaling during memory consolidation, which occurs within the first 1 h after learning (Ichinose et al., 2015; Musso et al., 2015). Blocking the CCHa2 neurons right after conditioning only for an hour impaired LTM (Figure 2B). The same blockade did not cause significant LTM impairment when applied at 22 h after conditioning (Figure 2C). Those results suggest that CCHa2 regulates the consolidation of LTM, at least through the post-learning activity in a specific time window.

**FIGURE 3:**
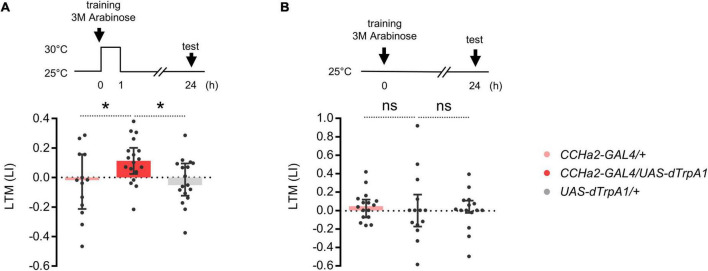
CCHa2 signal consolidates labile arabinose memory. **(A)** The thermal activation of CCHa2 neurons in *CCHa2-GAL4/UAS-dTrpA1* flies for an hour after training compels labile arabinose memory to be long-lasting. *p < 0.05 (Sidak’s multiple comparisons test, *N* = 13, 18, 17). **(B)** The arabinose memory is not observable after 24 h of training without thermal activation. ns, not significant (Sidak’s multiple comparisons test, *N* = 16, 14, 15).

We asked whether compelling CCHa2 cells after learning suffices for LTM formation. Flies can remember an odor paired with non-nutritive D-arabinose only for a short period of time (Burke and Waddell, 2011). We found that thermal activation of CCHa2 cells for an hour after conditioning turned otherwise labile arabinose memory into a long-lasting one (Figure 3A). No LTM was observed in the dTrpA1 uninduced control (Figure 3B). Since CCHa2 mediates sugar nutrient signals for growth control in larvae (Sano et al., 2015), we assumed it mediates delayed nutrient signals for appetitive memory consolidation.

### CCHamide-2 neurons in the brain

**FIGURE 4:**
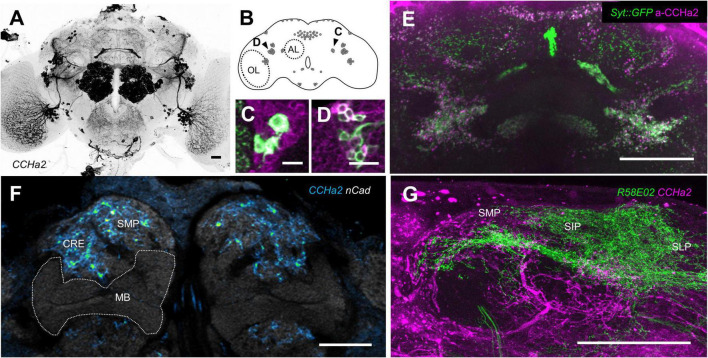
CCHa2 expression in the brain. **(A)** Brain expression of CCHa2 visualized in *CCHa2-GAL4/UAS-mCD8:GFP* flies. Scale bar, 20 μm. **(B)** Schematic representation of CCHa2 neurons in the brain. **(C,D)** CCHa2 expressing cells (green) in *CCHa2-GAL4/UAS-mCD8:GFP* flies colocalize with anti-CCHa2 antibody, suggestive of *bona fide* CCHa2 neurons labeled by the GAL4. Scale bar, 10 μm. **(E)** Colocalization of immunolabeled CCHa2 (magenta) and synaptic terminals of the CCHa2 neurons (green) in *CCHa2-GAL4/UAS-Syt:GFP* flies. Scale bar, 50 μm. **(F)** Absence of CCHa2 neurons in the mushroom body. In contrast, they innervate the surrounding neuropils. Neuropils are visualized by n-Cadherin (nCad) counter-staining. CRE: crepine, SMP: superior medial protocerebrum. Scale bar, 20 μm. **(G)** Potential interactions between CCHa2 neurons (magenta) and rewarding dopamine neurons (green) of *CCHa2-GAL4, R58E02-LexA/UAS-mCH8:RFP, LexAop-rCD2:GFP* flies in the superior neuropils. SIP: superior intermediate protocerebrum, SLP: superior lateral protocerebrum. Scale bar, 50 μm.

To map CCHa2 cells, we visualized the expression of *CCHa2-T2A-GAL4*. We found robust CCHa2 expression in the brain neurons (Figure 4A) in addition to previously reported periphery tissues (Li et al., 2013; Veenstra and Ida, 2014; Ren et al., 2015; Sano et al., 2015). Approximately 130 cells are labeled and are clustered into ca. ten cell types per hemisphere (Figure 4B). The GAL4 expression replicated the immunoreactivity of CCHa2, which labels mostly CCHa2 positive cells in the brain at used dilution (Supplementary Figure 1), at the cellular and synaptic resolution (Figures 4C–E). Those neurons innervated the antennal lobe, the optic lobe, and the gnathal ganglia (Figure 4A), implying sensory modulation by CCHa2. Notably, CCHa2 was absent from the mushroom body (Figure 4F), where appetitive memory resides upon learning. Meanwhile, CCHa2 profiles were enriched in the surrounding neuropils of the mushroom body, such as the superior medial protocerebrum or the crepine, where most mushroom body-associated neurons innervate (Figure 4F). Moreover, the CCHa2 processes projected near the dendritic profiles of rewarding dopamine neurons (Figure 4G), suggestive of their interactions. Therefore, the brain CCHa2 neurons could directly modulate the mushroom body-associated neurons to affect memory consolidation.

### CCHa2-R in a subset of protocerebrum anterior median neurons for long-term memory consolidation

**FIGURE 5:**
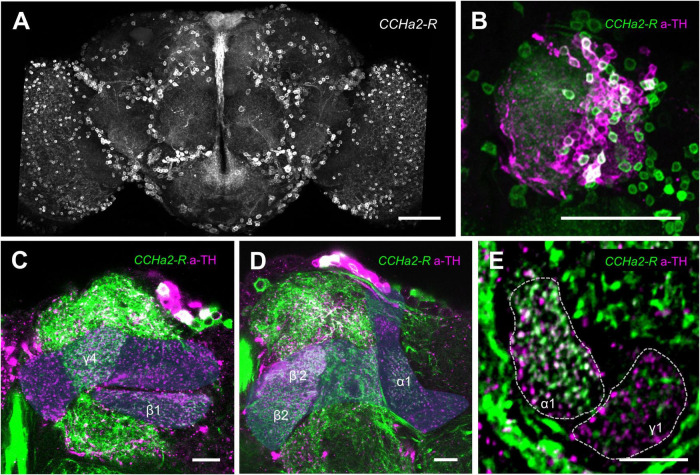
Appetitive dopamine neurons express CCHa2-R. **(A)** Brain expression of CCHa2-R visualized in *CCHa2-R-GAL4/UAS-mCD8:GFP* flies. Scale bar, 50 μm. **(B–D)** Double labeling of anti-tyrosine hydroxylase (magenta) and CCHa2 (green) expressing cells in *CCHa2-R-GAL4/UAS-mCD8:GFP* flies. Dozens of the protocerebrum anterior medial (PAM) cluster dopamine neuron nuclei are co-labeled, indicative of CCHa2-R expression in the PAM dopamine neurons **(B)**. Blue shades indicate the mushroom body. **(E)** Dopamine neurons innervating the α1 but not the γ1 compartment express CCHa2-R. Scale bars, 10 μm.

The post-learning activity of the LTM-inducing dopamine neurons regulates appetitive memory consolidation (Ichinose et al., 2015). We therefore hypothesized that CCHa2 exerts its memory function by directly regulating those dopamine neurons’ activity. We thus visualized the expression of CCHa2-R in the brain by using the *CCHa2-R-GAL4* (Sano et al., 2015). The receptor expression was abundant throughout the brain (Figure 5A), which included many of the protocerebrum anterior median (PAM) cluster dopamine neurons (Figure 5B). The CCHa2-R cells innervate to at least five MB compartments, including γ4, β’2, β2, β1, and α1 (Figures 5C,D). Among, colocalization with TH immunoreactivity was most evident in the α1 compartment (Figure 5E), suggesting that the CCHa2 targets a class of PAM neurons for appetitive LTM consolidation (Huetteroth et al., 2015; Yamagata et al., 2015). The sparse innervation in the γ1, in contrast, likely originates from non-dopamine neurons (Figure 5E).

**FIGURE 6:**
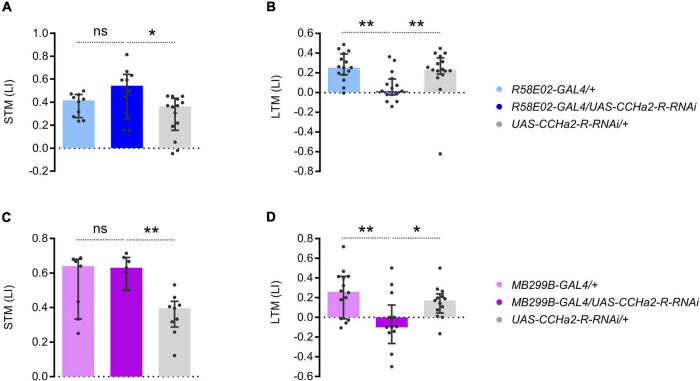
CCHa2-R in LTM-inducing dopamine neurons is required for LTM. **(A,B)** Cell type specific CCHa2-R silencing in the PAM neurons directed by *R58E02-GAL4*, showing LTM specific memory impairment. ns, not significant, **p < 0.01 (**A:** Holm–Sidak’s multiple comparisons test, *N* = 10, 10, 14, **B:** Dunn’s multiple comparisons test, *N* = 15, 15, 16). **(C,D)** PAM-α1 specific CCHa2-R silencing directed by *MB299B-GAL4*, showing LTM specific memory impairement. *p < 0.05 (**C:** Sidak’s multiple comparisons test, *N* = 7, 7, 9, **D:** Holm–Sidak’s multiple comparisons test, *N* = 14, 13, 13).

To examine the function of CCHa2-R in the PAM neurons, we down-regulated the receptor expression specifically in them. Expressing the short hairpin RNA of CCHa2-R in the entire PAM cluster by *R58E02-GAL4* abolished appetitive LTM but not STM (Figures 6A,B). Narrowing down the short hairpin RNA expression of CCHa2-R by *MB299B-GAL4*, which predominantly targets PAM-α1, still recapitulated the knock-down effect by *R58E02-GAL4* (Figures 6C,D). The more substantial RNAi effect than the CCHa2 mutant (Figure 1B) may imply additional off-target regulation of RNAi or other effective ligands that regulate CCHa2-R (Ida et al., 2012). Thus, CCHa2-R on the PAM-α1 neurons was critical for appetitive LTM. We concluded that CCHa2 mediates the delayed sugar effect to control the ongoing activity of PAM-α1 neurons during the memory consolidation phase for appetitive LTM.

## Discussion

Previous studies on CCHa2 have mainly focused on its role in feeding and energy homeostasis. Here, using *Drosophila*, we established the active involvement of CCHa2 in stabilizing appetitive memory. Precisely, it targeted a class of dopamine neurons for LTM in a time-dependent manner, suggesting the crucial role of CCHa2 and CCHa2-R in consolidating memory. Our results unveiled a novel peptidergic link between sugar metabolism and a cognitive process. The ecological relevance of the LTM regulation by CCHa2 is yet unclear. However, it could be adaptive that an endocrine system for energy homeostasis was related to the consolidation of food-related memory.

We have previously identified that dopamine neurons in the PAM cluster are central to processing sugar rewards (Burke et al., 2012; Liu et al., 2012). Two subsets of PAM neurons mediate parallel rewards for sweetness and nutrition to drive STM and LTM, respectively (Huetteroth et al., 2015; Yamagata et al., 2015). In particular, the post-learning activity of the PAM neurons for LTM, or a specific PAM-α1 class neuron, consolidates the nascent memory trace into LTM (Ichinose et al., 2015) depending on sugar nutrition. The PAM neuron forms a feedback loop with the mushroom body Kenyon cells and the output neurons (Aso et al., 2014; Li et al., 2020). While the feedback loop is suitable to retain the circuit activity (Ichinose et al., 2015), any signals that drive the activity remained elusive. Considering the narrow time window of the requirement for the CCHa2 neurons (Figures 2, 3), CCHa2 might be a signal to initiate and maintain the feedback circuit activity for memory consolidation.

Musso et al. (2017) demonstrated that sugar experiences without caloric inputs subsequently decrease the reward value of sugar, so-called caloric frustration memory. Since CCHa2 mutant flies cannot detect sugar nutrition, the caloric frustration memory can explain, at least partially, the mutant effect of CCHa2, too. If this is the case, CCHa2 should counteract the sweetness signal attenuating subsequent sugar responses in the PAM neurons. Such an integrative process of competitive sugar sweetness and nutrient signals within PAM neurons would be an interesting perspective of the current study.

Supplementing nutrient sugar after non-nutritive sugar learning turns the labile STM into LTM (Musso et al., 2015), indicative of the delayed sugar effect for LTM consolidation. Previously, a class of aversive dopamine neurons, PPL1-γ1pedc (a.k.a. MB-MP1) (Krashes et al., 2009; Aso et al., 2012), was allocated to the delayed effect. The cell class exhibits sustained activity after learning depending on sugar nutrition (Musso et al., 2015). The activity upregulates energy flux in the mushroom body to gate memory consolidation (Plaçais et al., 2017). The functional correlation between the PPL1- and the PAM-mediated consolidation mechanisms is yet unresolved, though the overlapping narrow time windows imply their functional interplay. Clarifying the finer temporal requirement of the CCHa2 neurons, including the early consolidation phase in the first half an hour after learning (Musso et al., 2015), may help unveil the complex interaction between the two MB-associated local circuitries.

The signaling source of CCHa2 that mediates sugar nutrition to the PAM neurons remains elusive. Our data suggest that any of the CCHa2 neurons that innervate the superior neuropils or the crepine (Figure 4F) are the prime candidate as an upstream mediator of the nutrient signal. Those candidate neurons may interact with the PAM-α1 dendrites directly (Figure 4G) to regulate the neuronal activity toward memory consolidation. Alternatively, peripheral CCHa2 may act directly onto the PAM neurons. Because of the preferential expression of CCHa2 in the peripheral tissue (Li et al., 2013; Veenstra and Ida, 2014; Hung et al., 2020) and its permeability to the blood–brain barrier (Sano et al., 2015), former studies assumed peripheral-brain endocrine action of CCHa2 (Ren et al., 2015; Sano et al., 2015, 2022). In mammals, the gut-brain axis for regulating reward, learning, and memory is prevailing (Han et al., 2018; Suarez et al., 2018; Davis et al., 2020), suggesting the importance of intestinal health for proper cognition. The intestinal microbiome is also associated with various mood and mental disorders (Valles-Colomer et al., 2019; McGuinness et al., 2022), leading to new therapeutic strategies. In flies, such a gut-brain axis is established for feeding regulation (Kobler et al., 2020; Titos and Rogulja, 2020; Kim et al., 2021), aggression (Jia et al., 2021), and behavioral switch between courtship and feeding (Lin et al., 2022), providing valuable prototypes of the mammalian system to identify key signaling molecules and their cellular actions. However, critical evidence supporting the gut-brain axis in learning and memory is scarce (but see Silva et al., 2021). Future studies should thus follow up on whether CCHa2 comes from peripheral tissues or brain neurons expressing it to elaborate circuit dynamism underlying memory consolidation.

In this study, we have identified the as-yet-unknown cognitive role of CCHa2 and CCHa2-R. Since the bombesin receptor subtype 3, the mammalian ortholog of CCHa2-R, is also implicated in memory consolidation (Flood and Morley, 1988; Shumyatsky et al., 2002; Roesler et al., 2004; Ghanbari et al., 2018; Melzer et al., 2021), our findings may further reinforce the evolutionarily conserved function of the CCHa2 system. Moreover, it is noteworthy that the bombesin receptor is involved in the etiology of Alzheimer’s disease (Roesler et al., 2007). The notion is supported by the abnormal receptor signaling observed in the patient’s fibroblasts and a transgenic mouse model of Alzheimer’s disease (Ito et al., 1994; Huang et al., 2005). In addition, drugs acting at the receptor enhance memory and ameliorate cognitive dysfunction in animal disease models (Roesler and Schwartsmann, 2012). *Drosophila* models for Alzheimer’s disease rely on the aggressive fibril formation through γ- and β-secretase cleavage of the amyloid-beta precursor peptide (Tsuda and Lim, 2018), which predominantly affects LTM (Goguel et al., 2011; Bourdet et al., 2015; Rieche et al., 2018; Silva et al., 2020). The potential role of CCHa2 signaling on the pathogenesis of neurodegenerative diseases could be an interesting future path for the present study.

## Materials and methods

### Flies

Canton-S was used as a wild-type strain. *w;R58E02-GAL4* (Jenett et al., 2012; Liu et al., 2012), *w;R58E02-LexA* (Liu et al., 2012) *w;CCHa2-GAL4* (Kondo et al., 2020), *w;CCHa2-R-GAL4:p65* (Sano et al., 2015), *w;MB299B-GAL4* (Aso et al., 2014), *w;TH-GAL4* (Friggi-Grelin et al., 2003), *w;UAS-Shibire[ts1]* (pJFRC100-20XUAS-TTS-Shibire-ts1-p10) (Pfeiffer et al., 2012), *w;UAS-dTrpA1* (Hamada et al., 2008), *w;UAS-CCHa2-R-shRNA #24* (Sano et al., 2015), *w;UAS-syt.eGFP* (BDSC #6926), *CCHa2^CR–1^ and CCHa2^CR–3^* (Sano et al., 2015) were previously described. Flies were raised at 24°C with 12:12 LD cycle. Knock-down flies were prepared as the F1 progeny of the crosses between females of *w;R58E02-GAL4* or *w;MB299-GAL4* or *w* and males of UAS effectors or CS (Figure 5). The F1 progeny was raised at 24°C, aged 3-12 d after eclosion before experiments. For immunohistochemistry, a female reporter strain *w;UAS-mCD8:GFP* (Figures 4, 5), *w;UAS-syt.eGFP* (Figure 4E) or *w;LexAop-rCD2:GFP;UAS-mCD8:RFP* (Figure 4G) was crossed to male GAL4 drivers or a reporter strain, *w;CCHa2-R-GAL4:p65, w;R58E02-GAL4, w;R58E02-LexA,CCHa2-GAL4, w;TH-GAL4*, or *w;CCHa2-GAL4*. Flies used for whole-mount immunohistochemistry were aged 3–10 days after eclosion. A complete list of the fly crosses for behavioral experiments is listed in Supplementary Table 1.

### Behavioral assays

The conditioning and testing protocols were as described previously (Yamagata et al., 2015, 2016). Briefly, a group of approximately 50 flies in a training tube alternately received octan-3-ol (3OCT; Merck) and 4-methylcyclohexanol (4MCH; Sigma-Aldrich) for 1 min in a constant air stream with or without dried 2M sucrose paper (Figures 1, 5). For the temperature shift protocol (Figure 2), flies received two odors and dried sugar alternately either at room temperature or 33°C in a heat box. For the post-learning activation protocol, flies were trained with 3M D-arabinose paper (Figure 3). Then the conditioned response of the trained flies was measured. Flies were given a choice between sugar paired (CS +) and unpaired (CS-) odors for 2 min in a T maze immediately after training (Figures 1A, 5A,C). Flies were kept in a vial with water-soaked paper for 24 h before testing LTM. For temperature shift and post-learning activation protocols, flies were incubated in a heat box at a defined time and temperature. Odors were diluted to 10% in the paraffin oil and placed in a cup with a diameter of 3 mm (OCT) or 5 mm (MCH). A learning index was calculated by taking the mean preference of the two reciprocally trained groups. Half of the trained groups received reinforcement with the first presented odor and the other half with the second odor to cancel the effect of the reinforcement order.

### Brain dissection and immunohistochemistry

Dissection of fly brains was performed as previously described (Kondo et al., 2020) with minor modifications. Brains of female flies were dissected in PBS, pre-fixed in 1% paraformaldehyde (PFA) in PBS on the ice for up to 30 min, then fixed in 2% PFA in PBS for 1 h at room temperature. Fixed brains were washed in PBT (0.1% Triton X-100 in PBS) for 3 × 10 min. Immunostaining was performed by Kondo et al. (2020). The following primary antibodies were used at the indicated dilution: rabbit anti-GFP (1:1,000; Invitrogen; A11122), mouse anti-TH (1:100; ImmunoStar Inc.; 22941), rabbit anti-CCHa2 (1:1,000) (Ida et al., 2012), and rat anti-N-cadherin (DN-EX #8; 1:100; Developmental Studies Hybridoma Bank). The following secondary antibodies were used at the indicated dilution: AlexaFluor-488 goat anti-rabbit (1:1,000; Invitrogen; A11034), Cy3 goat anti-rabbit (1:200; Jackson Labs), Cy3 goat anti-rat (1:200; Jackson Labs), and AlexaFluor-568 goat anti-mouse (1:1,000; Invitrogen; 11004). 86% Glycerol was used as a mounting medium, and either native or immunostained fluorescence was imaged.

### Confocal imaging

Imaging was performed on the Olympus FV1200 confocal microscope with GaAsP sensors. A 30x/1.05 silicone immersion objective (UPLSAPO30XS, Olympus) (Figures 4A, 5A) or a 60x/1.42 oil immersion objective (PLAPON60XO, Olympus) (Figures 4C–G, 5B–E) was used for scanning specific regions of interest. Confocal stacks were analyzed with the open-source software Image-J (National Institute of Health) and Fiji (Schindelin et al., 2012). Where appropriate, 2D/3D image deconvolution was applied using Diffraction PSF 3D and Parallel Iterative Deconvolution plugins in Image-J.

### Statistics

Statistics were performed by Prism5 (Graphpad). For the data points that did not violate the assumption of normality and homogeneity of variance (D’Agostino and Brown-Forsythe test), parametric statistics were applied. The data points that were significantly different from the normal distribution were analyzed with non-parametric statistics. *A priori* power analysis has been made with G*Power (Faul et al., 2007, 2009) to estimate the required N to achieve power (1-β) greater than 0.8 (Cohen, 1988; Supplementary Table 1). Based on the standard practice in the field, alpha and effect size were set to 0.05 and 0.5, respectively. The actual effect size was also calculated *post hoc* and listed in Supplementary Table 1.

## Data availability statement

The original contributions presented in this study are included in the article/Supplementary material, further inquiries can be directed to the corresponding author.

## Author contributions

NY and HT: conceptualization, writing—review and editing, and supervision. NY: methodology, formal analysis, writing—original draft preparation, and visualization. NY, YI, and HW: investigation. SK and HS: genetic tool. All authors contributed to the article and approved the submitted version.

## Supplementary material

Supplementary Figure 1 Antibody staining of CCHa2 in the wild type (upper) and CCHa2 CR1 mutant (lower) brains. The a-CCHa2 signal was largely diminished in the mutant brain except for a small fraction of cells in the medial protocerebrum, the optic lobe, the tritocerebrum, and the gnathal ganglia, suggestive of reasonable specificity of the antibody. No apparent pattern difference was observed in the two staining conditions, though there was less background signal in the lower dilution.

## References

[B1] Aso Y. Hattori D. Yu Y. Johnston R. M. Iyer N. A. Ngo T.-T. B. (2014). The neuronal architecture of the mushroom body provides a logic for associative learning. *Elife* 3:e04577. 10.7554/eLife.04577 25535793 PMC4273437

[B2] Aso Y. Herb A. Ogueta M. Siwanowicz I. Templier T. Friedrich A. B. (2012). Three dopamine pathways induce aversive odor memories with different stability. *PLoS Genet.* 8:e1002768. 10.1371/journal.pgen.1002768 22807684 PMC3395599

[B3] Bourdet I. Lampin-Saint-Amaux A. Preat T. Goguel V. (2015). Amyloid-β peptide exacerbates the memory deficit caused by amyloid precursor protein loss-of-function in *Drosophila*. *PLoS One* 10:e0135741. 10.1371/journal.pone.0135741 26274614 PMC4537105

[B4] Burke C. J. Waddell S. (2011). Remembering nutrient quality of sugar in *Drosophila*. *Curr. Biol.* 21 746–750. 10.1016/j.cub.2011.03.032 21514159 PMC3094154

[B5] Burke C. J. Huetteroth W. Owald D. Perisse E. Krashes M. J. Das G. (2012). Layered reward signalling through octopamine and dopamine in *Drosophila*. *Nature* 492 433–437. 10.1038/nature11614 23103875 PMC3528794

[B6] Capriotti N. Ianowski J. P. Gioino P. Ons S. (2019). The neuropeptide CCHamide2 regulates diuresis in the Chagas disease vector *Rhodnius prolixus*. *J. Exp. Biol.* 222(Pt 10):jeb203000. 10.1242/jeb.203000 31053646

[B7] Chambers D. B. Androschuk A. Rosenfelt C. Langer S. Harding M. Bolduc F. V. (2015). Insulin signaling is acutely required for long-term memory in *Drosophila*. *Front. Neural Circuits* 9:8. 10.3389/fncir.2015.00008 25805973 PMC4354381

[B8] Christie A. E. (2015). Neuropeptide discovery in *Symphylella vulgaris* (Myriapoda, Symphyla): In silico prediction of the first myriapod peptidome. *Gen. Comp. Endocrinol.* 223 73–86. 10.1016/j.ygcen.2015.09.021 26407502

[B9] Chung B. Y. Ro J. Hutter S. A. Miller K. M. Guduguntla L. S. Kondo S. (2017). *Drosophila* neuropeptide F signaling independently regulates feeding and sleep-wake behavior. *Cell Rep.* 19 2441–2450. 10.1016/j.celrep.2017.05.085 28636933 PMC5536846

[B10] Cohen J. (1988). *Statistical power analysis for the behavioral sciences*, 2nd Edn. Hillsdale, NJ: Lawrence Erlbaum Associates.

[B11] Davis E. A. Wald H. S. Suarez A. N. Zubcevic J. Liu C. M. Cortella A. M. (2020). Ghrelin signaling affects feeding behavior, metabolism, and memory through the vagus nerve. *Curr. Biol.* 30 4510–4518.e6. 10.1016/j.cub.2020.08.069 32946754 PMC7674191

[B12] Eschment M. Franz H. R. Güllü N. Hölscher L. G. Huh K.-E. Widmann A. (2020). Insulin signaling represents a gating mechanism between different memory phases in *Drosophila* larvae. *PLoS Genet.* 16:e1009064. 10.1371/journal.pgen.1009064 33104728 PMC7644093

[B13] Faul F. Erdfelder E. Buchner A. Lang A. G. (2009). Statistical power analyses using G*Power 3.1: Tests for correlation and regression analyses. *Behav. Res. Methods* 41 1149–1160. 10.3758/brm.41.4.1149 19897823

[B14] Faul F. Erdfelder E. Lang A. G. Buchner A. (2007). G*Power 3: A flexible statistical power analysis program for the social, behavioral, and biomedical sciences. *Behav. Res. Methods* 39 175–191. 10.3758/bf03193146 17695343

[B15] Feng K.-L. Weng J.-Y. Chen C.-C. Abubaker M. B. Lin H.-W. Charng C.-C. (2021). Neuropeptide F inhibits dopamine neuron interference of long-term memory consolidation in *Drosophila*. *iScience* 24:103506. 10.1016/j.isci.2021.103506 34934925 PMC8661542

[B16] Flood J. F. Morley J. E. (1988). Effects of bombesin and gastrin-releasing peptide on memory processing. *Brain Res.* 460 314–322. 10.1016/0006-8993(88)90375-7 3224263

[B17] Friggi-Grelin F. Coulom H. Meller M. Gomez D. Hirsh J. Birman S. (2003). Targeted gene expression in *Drosophila* dopaminergic cells using regulatory sequences from tyrosine hydroxylase. *J. Neurobiol.* 54 618–627. 10.1002/neu.10185 12555273

[B18] Fujita M. Tanimura T. (2011). *Drosophila* evaluates and learns the nutritional value of sugars. *Curr. Biol.* 21 751–755. 10.1016/j.cub.2011.03.058 21514154

[B19] Fujiwara Y. Hermann-Luibl C. Katsura M. Sekiguchi M. Ida T. Helfrich-Förster C. (2018). The CCHamide1 neuropeptide expressed in the anterior dorsal neuron 1 conveys a circadian signal to the ventral lateral neurons in *Drosophila melanogaster*. *Front. Physiol.* 9:1276. 10.3389/fphys.2018.01276 30246807 PMC6139358

[B20] Ghanbari A. Moradi Kor N. Rashidy-Pour A. (2018). Bombesin-induced enhancement of memory consolidation in male and female rat pups: Role of glutamatergic and dopaminergic systems. *Neuropeptides* 70 101–106. 10.1016/j.npep.2018.05.011 29880391

[B21] Goguel V. Belair A.-L. Ayaz D. Lampin-Saint-Amaux A. Scaplehorn N. Hassan B. A. (2011). *Drosophila* amyloid precursor protein-like is required for long-term memory. *J. Neurosci.* 31 1032–1037. 10.1523/jneurosci.2896-10.2011 21248128 PMC6632943

[B22] Hamada F. N. Rosenzweig M. Kang K. Pulver S. R. Ghezzi A. Jegla T. J. (2008). An internal thermal sensor controlling temperature preference in *Drosophila*. *Nature* 454 217–220. 10.1038/nature07001 18548007 PMC2730888

[B23] Han W. Tellez L. A. Perkins M. H. Perez I. O. Qu T. Ferreira J. (2018). A neural circuit for gut-induced reward. *Cell* 175 665–678.e23. 10.1016/j.cell.2018.08.049 30245012 PMC6195474

[B24] Hansen K. K. Hauser F. Williamson M. Weber S. B. Grimmelikhuijzen C. J. (2011). The *Drosophila* genes CG14593 and CG30106 code for G-protein-coupled receptors specifically activated by the neuropeptides CCHamide-1 and CCHamide-2. *Biochem. Biophys. Res. Commun.* 404 184–189. 10.1016/j.bbrc.2010.11.089 21110953

[B25] Havula E. Ghazanfar S. Lamichane N. Francis D. Hasygar K. Liu Y. (2022). Genetic variation of macronutrient tolerance in *Drosophila melanogaster*. *Nat. Commun.* 13:1637. 10.1038/s41467-022-29183-x 35347148 PMC8960806

[B26] Huang H. M. Chen H. L. Xu H. Gibson G. E. (2005). Modification of endoplasmic reticulum Ca2+ stores by select oxidants produces changes reminiscent of those in cells from patients with Alzheimer disease. *Free Radic. Biol. Med.* 39 979–989. 10.1016/j.freeradbiomed.2005.05.017 16198225

[B27] Huetteroth W. Perisse E. Lin S. Klappenbach M. Burke C. Waddell S. (2015). Sweet taste and nutrient value subdivide rewarding dopaminergic neurons in *Drosophila*. *Curr. Biol.* 25 751–758. 10.1016/j.cub.2015.01.036 25728694 PMC4372253

[B28] Hung R.-J. Hu Y. Kirchner R. Liu Y. Xu C. Comjean A. (2020). A cell atlas of the adult *Drosophila* midgut. *Proc. Natl. Acad. Sci. U.S.A.* 117 1514–1523. 10.1073/pnas.1916820117 31915294 PMC6983450

[B29] Ichinose T. Aso Y. Yamagata N. Abe A. Rubin G. M. Tanimoto H. (2015). Reward signal in a recurrent circuit drives appetitive long-term memory formation. *Elife* 4:e10719. 10.7554/eLife.10719 26573957 PMC4643015

[B30] Ida T. Takahashi T. Tominaga H. Sato T. Sano H. Kume K. (2012). Isolation of the bioactive peptides CCHamide-1 and CCHamide-2 from *Drosophila* and their putative role in appetite regulation as ligands for G protein-coupled receptors. *Front. Endocrinol. (Lausanne)* 3:177. 10.3389/fendo.2012.00177 23293632 PMC3533232

[B31] Ito E. Oka K. Etcheberrigaray R. Nelson T. J. McPhie D. L. Tofel-Grehl B. (1994). Internal Ca2+ mobilization is altered in fibroblasts from patients with Alzheimer disease. *Proc. Natl. Acad. Sci. U.S.A.* 91 534–538. 10.1073/pnas.91.2.534 8290560 PMC42983

[B32] Jayakumar S. Richhariya S. Deb B. K. Hasan G. (2018). A multicomponent neuronal response encodes the larval decision to pupariate upon amino acid starvation. *J. Neurosci.* 38 10202–10219. 10.1523/jneurosci.1163-18.2018 30301757 PMC6246885

[B33] Jenett A. Rubin G. M. Ngo T. T. Shepherd D. Murphy C. Dionne H. (2012). A GAL4-driver line resource for *Drosophila* neurobiology. *Cell Rep.* 2 991–1001. 10.1016/j.celrep.2012.09.011 23063364 PMC3515021

[B34] Jia Y. Jin S. Hu K. Geng L. Han C. Kang R. (2021). Gut microbiome modulates *Drosophila* aggression through octopamine signaling. *Nat. Commun.* 12:2698. 10.1038/s41467-021-23041-y 33976215 PMC8113466

[B35] Jin K. Wilson K. A. Beck J. N. Nelson C. S. Brownridge G. W. III Harrison B. R. (2020). Genetic and metabolomic architecture of variation in diet restriction-mediated lifespan extension in *Drosophila*. *PLoS Genet.* 16:e1008835. 10.1371/journal.pgen.1008835 32644988 PMC7347105

[B36] Kim B. Kanai M. I. Oh Y. Kyung M. Kim E.-K. Jang I.-H. (2021). Response of the microbiome–gut–brain axis in *Drosophila* to amino acid deficit. *Nature* 593 570–574. 10.1038/s41586-021-03522-2 33953396

[B37] Kobler J. M. Rodriguez Jimenez F. J. Petcu I. Grunwald Kadow I. C. (2020). Immune receptor signaling and the mushroom body mediate post-ingestion pathogen avoidance. *Curr. Biol.* 30 4693–4709.e3. 10.1016/j.cub.2020.09.022 33007248

[B38] Kondo S. Takahashi T. Yamagata N. Imanishi Y. Katow H. Hiramatsu S. (2020). Neurochemical organization of the *Drosophila* brain visualized by endogenously tagged neurotransmitter receptors. *Cell Rep.* 30 284–297.e5. 10.1016/j.celrep.2019.12.018 31914394

[B39] Krashes M. J. DasGupta S. Vreede A. White B. Armstrong J. D. Waddell S. (2009). A neural circuit mechanism integrating motivational state with memory expression in *Drosophila*. *Cell* 139 416–427. 10.1016/j.cell.2009.08.035 19837040 PMC2780032

[B40] Landayan D. Wang B. P. Zhou J. Wolf F. W. (2021). Thirst interneurons that promote water seeking and limit feeding behavior in *Drosophila*. *Elife* 10:e66286. 10.7554/eLife.66286 34018925 PMC8139827

[B41] Li F. Lindsey J. W. Marin E. C. Otto N. Dreher M. Dempsey G. (2020). The connectome of the adult *Drosophila* mushroom body provides insights into function. *Elife* 9:e62576. 10.7554/eLife.62576 33315010 PMC7909955

[B42] Li S. Torre-Muruzabal T. Søgaard K. C. Ren G. R. Hauser F. Engelsen S. M. (2013). Expression patterns of the *Drosophila* neuropeptide CCHamide-2 and its receptor may suggest hormonal signaling from the gut to the brain. *PLoS One* 8:e76131. 10.1371/journal.pone.0076131 24098432 PMC3788761

[B43] Lin H. H. Kuang M. C. Hossain I. Xuan Y. Beebe L. Shepherd A. K. (2022). A nutrient-specific gut hormone arbitrates between courtship and feeding. *Nature* 602 632–638. 10.1038/s41586-022-04408-7 35140404 PMC9271372

[B44] Lin S. Senapati B. Tsao C. H. (2019). Neural basis of hunger-driven behaviour in *Drosophila*. *Open Biol.* 9:180259. 10.1098/rsob.180259 30914005 PMC6451361

[B45] Liu C. Plaçais P.-Y. Yamagata N. Pfeiffer B. D. Aso Y. Friedrich A. B. (2012). A subset of dopamine neurons signals reward for odour memory in *Drosophila*. *Nature* 488 512–516. 10.1038/nature11304 22810589

[B46] McGinnis J. P. Jiang H. Agha M. A. Sanchez C. P. Lange J. Yu Z. (2016). Immediate perception of a reward is distinct from the reward’s long-term salience. *Elife* 5:e22283. 10.7554/eLife.22283 28005005 PMC5243026

[B47] McGuinness A. J. Davis J. A. Dawson S. L. Loughman A. Collier F. O’Hely M. (2022). A systematic review of gut microbiota composition in observational studies of major depressive disorder, bipolar disorder and schizophrenia. *Mol. Psychiatry* 27 1920–1935. 10.1038/s41380-022-01456-3 35194166 PMC9126816

[B48] Melzer S. Newmark E. R. Mizuno G. O. Hyun M. Philson A. C. Quiroli E. (2021). Bombesin-like peptide recruits disinhibitory cortical circuits and enhances fear memories. *Cell* 184 5622–5634.e25. 10.1016/j.cell.2021.09.013 34610277 PMC8556345

[B49] Mery F. Kawecki T. J. (2005). A cost of long-term memory in *Drosophila*. *Science* 308 1148–1148. 10.1126/science.1111331 15905396

[B50] Musso P.-Y. Lampin-Saint-Amaux A. Tchenio P. Preat T. (2017). Ingestion of artificial sweeteners leads to caloric frustration memory in *Drosophila*. *Nat. Commun.* 8:1803. 10.1038/s41467-017-01989-0 29180783 PMC5703724

[B51] Musso P.-Y. Tchenio P. Preat T. (2015). Delayed dopamine signaling of energy level builds appetitive long-term memory in *Drosophila*. *Cell Rep.* 10 1023–1031. 10.1016/j.celrep.2015.01.036 25704807

[B52] Nässel D. R. Pauls D. Huetteroth W. (2019). Neuropeptides in modulation of *Drosophila* behavior: How to get a grip on their pleiotropic actions. *Curr. Opin. Insect Sci.* 36 1–8. 10.1016/j.cois.2019.03.002 31280184

[B53] Nguyen T. V. Rotllant G. E. Cummins S. F. Elizur A. Ventura T. (2018). Insights into sexual maturation and reproduction in the Norway Lobster (*Nephrops norvegicus*) via in silico prediction and characterization of neuropeptides and G protein-coupled receptors. *Front. Endocrinol. (Lausanne)* 9:430. 10.3389/fendo.2018.00430 30100897 PMC6073857

[B54] Ohki-Hamazaki H. Watase K. Yamamoto K. Ogura H. Yamano M. Yamada K. (1997). Mice lacking bombesin receptor subtype-3 develop metabolic defects and obesity. *Nature* 390 165–169. 10.1038/36568 9367152

[B55] Pfeiffer B. D. Truman J. W. Rubin G. M. (2012). Using translational enhancers to increase transgene expression in *Drosophila*. *Proc. Natl. Acad. Sci. U.S.A.* 109 6626–6631. 10.1073/pnas.1204520109 22493255 PMC3340069

[B56] Piñol R. A. Zahler S. H. Li C. Saha A. Tan B. K. Škop V. (2018). Brs3 neurons in the mouse dorsomedial hypothalamus regulate body temperature, energy expenditure, and heart rate, but not food intake. *Nat. Neurosci.* 21 1530–1540. 10.1038/s41593-018-0249-3 30349101 PMC6203600

[B57] Plaçais P. Y. Preat T. (2013). To favor survival under food shortage, the brain disables costly memory. *Science* 339 440–442. 10.1126/science.1226018 23349289

[B58] Plaçais P.-Y. de Tredern É Scheunemann L. Trannoy S. Goguel V. Han K.-A. (2017). Upregulated energy metabolism in the *Drosophila* mushroom body is the trigger for long-term memory. *Nat. Commun.* 8:15510. 10.1038/ncomms15510 28580949 PMC5465319

[B59] Ren G. R. Hauser F. Rewitz K. F. Kondo S. Engelbrecht A. F. Didriksen A. K. (2015). CCHamide-2 Is an orexigenic brain-gut peptide in *Drosophila*. *PLoS One* 10:e0133017. 10.1371/journal.pone.0133017 26168160 PMC4500396

[B60] Rieche F. Carmine-Simmen K. Poeck B. Kretzschmar D. Strauss R. (2018). *Drosophila* full-length amyloid precursor protein is required for visual working memory and prevents age-related memory impairment. *Curr. Biol.* 28 817–823.e3. 10.1016/j.cub.2018.01.077 29478851 PMC5840017

[B61] Roesler R. Schwartsmann G. (2012). Gastrin-releasing peptide receptors in the central nervous system: Role in brain function and as a drug target. *Front. Endocrinol. (Lausanne)* 3:159. 10.3389/fendo.2012.00159 23251133 PMC3523293

[B62] Roesler R. Lessa D. Venturella R. Vianna M. R. Luft T. Henriques J. A. (2004). Bombesin/gastrin-releasing peptide receptors in the basolateral amygdala regulate memory consolidation. *Eur. J. Neurosci.* 19 1041–1045. 10.1111/j.0953-816x.2004.03175.x 15009151

[B63] Roesler R. Luft T. Schwartsmann G. (2007). Targeting the gastrin-releasing peptide receptor pathway to treat cognitive dysfunctionassociated with Alzheimer’s disease. *Dement. Neuropsychol.* 1 118–123. 10.1590/s1980-57642008dn10200002 29213377 PMC5619558

[B64] Rohwedder A. Selcho M. Chassot B. Thum A. S. (2015). Neuropeptide F neurons modulate sugar reward during associative olfactory learning of *Drosophila* larvae. *J. Comp. Neurol.* 523 2637–2664. 10.1002/cne.23873 26234537

[B65] Roller L. Yamanaka N. Watanabe K. Daubnerová I. Žitòan D. Kataoka H. (2008). The unique evolution of neuropeptide genes in the silkworm *Bombyx mori*. *Insect Biochem. Mol. Biol.* 38 1147–1157. 10.1016/j.ibmb.2008.04.009 19280707

[B66] Rulifson E. J. Kim S. K. Nusse R. (2002). Ablation of insulin-producing neurons in flies: Growth and diabetic phenotypes. *Science* 296 1118–1120. 10.1126/science.1070058 12004130

[B67] Sano H. (2015). Coupling of growth to nutritional status: The role of novel periphery-to-brain signaling by the CCHa2 peptide in *Drosophila melanogaster*. *Fly* 9 183–187. 10.1080/19336934.2016.1162361 26980588 PMC4862427

[B68] Sano H. Nakamura A. Texada M. J. Truman J. W. Ishimoto H. Kamikouchi A. (2015). The nutrient-responsive hormone CCHamide-2 controls growth by regulating insulin-like peptides in the brain of *Drosophila melanogaster*. *PLoS Genet.* 11:e1005209. 10.1371/journal.pgen.1005209 26020940 PMC4447355

[B69] Sano H. Nakamura A. Yamane M. Niwa H. Nishimura T. Araki K. (2022). The polyol pathway is an evolutionarily conserved system for sensing glucose uptake. *PLoS Biol.* 20:e3001678. 10.1371/journal.pbio.3001678 35687590 PMC9223304

[B70] Schindelin J. Arganda-Carreras I. Frise E. Kaynig V. Longair M. Pietzsch T. (2012). Fiji: An open-source platform for biological-image analysis. *Nat. Methods* 9 676–682. 10.1038/nmeth.2019 22743772 PMC3855844

[B71] Sgammeglia N. Sprecher S. G. (2022). Interplay between metabolic energy regulation and memory pathways in *Drosophila*. *Trends Neurosci.* 45 539–549. 10.1016/j.tins.2022.04.007 35597687

[B72] Shahid S. Shi Y. Yang C. Li J. Ali M. Y. Smagghe G. (2021). CCHamide2-receptor regulates feeding behavior in the pea aphid, *Acyrthosiphon pisum*. *Peptides* 143:170596. 10.1016/j.peptides.2021.170596 34118362

[B73] Shumyatsky G. P. Tsvetkov E. Malleret G. Vronskaya S. Hatton M. Hampton L. (2002). Identification of a signaling network in lateral nucleus of amygdala important for inhibiting memory specifically related to learned fear. *Cell* 111 905–918. 10.1016/S0092-8674(02)01116-9 12526815

[B74] Silva B. Niehage C. Maglione M. Hoflack B. Sigrist S. J. Wassmer T. (2020). Interactions between amyloid precursor protein-like (APPL) and MAGUK scaffolding proteins contribute to appetitive long-term memory in *Drosophila melanogaster*. *J. Neurogenet.* 34 92–105. 10.1080/01677063.2020.1712597 31965876

[B75] Silva V. Palacios-Muñoz A. Okray Z. Adair K. L. Waddell S. Douglas A. E. (2021). The impact of the gut microbiome on memory and sleep in *Drosophila*. *J. Exp. Biol.* 224(Pt 3):jeb233619. 10.1242/jeb.233619 33376141 PMC7875489

[B76] Suarez A. N. Hsu T. M. Liu C. M. Noble E. E. Cortella A. M. Nakamoto E. M. (2018). Gut vagal sensory signaling regulates hippocampus function through multi-order pathways. *Nat. Commun.* 9:2181. 10.1038/s41467-018-04639-1 29872139 PMC5988686

[B77] Thiel D. Bauknecht P. Jékely G. Hejnol A. (2019). A nemertean excitatory peptide/CCHamide regulates ciliary swimming in the larvae of *Lineus longissimus*. *Front. Zool.* 16:28. 10.1186/s12983-019-0326-9 31333754 PMC6617912

[B78] Titos I. Rogulja D. (2020). A gut-secreted peptide controls arousability through modulation of dopaminergic neurons in the brain. *bioRxiv* [Preprint]. bioRxiv 2020.2008.2031.275552. 10.1101/2020.08.31.275552

[B79] Toullec J. Y. Corre E. Mandon P. Gonzalez-Aravena M. Ollivaux C. Lee C. Y. (2017). Characterization of the neuropeptidome of a Southern ocean decapod, the Antarctic shrimp *Chorismus antarcticus*: Focusing on a new decapod ITP-like peptide belonging to the CHH peptide family. *Gen. Comp. Endocrinol.* 252 60–78. 10.1016/j.ygcen.2017.07.015 28728885

[B80] Tsao C. H. Chen C. C. Lin C. H. Yang H. Y. Lin S. (2018). *Drosophila* mushroom bodies integrate hunger and satiety signals to control innate food-seeking behavior. *Elife* 7:e35264. 10.7554/eLife.35264 29547121 PMC5910021

[B81] Tsuda L. Lim Y. M. (2018). Alzheimer’s disease model system using *Drosophila*. *Adv. Exp. Med. Biol.* 1076 25–40. 10.1007/978-981-13-0529-0_3 29951813

[B82] Valles-Colomer M. Falony G. Darzi Y. Tigchelaar E. F. Wang J. Tito R. Y. (2019). The neuroactive potential of the human gut microbiota in quality of life and depression. *Nat. Microbiol.* 4 623–632. 10.1038/s41564-018-0337-x 30718848

[B83] Veenstra J. A. (2016). Neuropeptide evolution: Chelicerate neurohormone and neuropeptide genes may reflect one or more whole genome duplications. *Gen. Comp. Endocrinol.* 229 41–55. 10.1016/j.ygcen.2015.11.019 26928473

[B84] Veenstra J. A. Ida T. (2014). More *Drosophila* enteroendocrine peptides: Orcokinin B and the CCHamides 1 and 2. *Cell Tissue Res.* 357 607–621. 10.1007/s00441-014-1880-2 24850274

[B85] Yamagata N. Hiroi M. Kondo S. Abe A. Tanimoto H. (2016). Suppression of dopamine neurons mediates reward. *PLoS Biol.* 14:e1002586. 10.1371/journal.pbio.1002586 27997541 PMC5172549

[B86] Yamagata N. Ichinose T. Aso Y. Plaçais P. Y. Friedrich A. B. Sima R. J. (2015). Distinct dopamine neurons mediate reward signals for short- and long-term memories. *Proc. Natl. Acad. Sci. U.S.A.* 112 578–583. 10.1073/pnas.1421930112 25548178 PMC4299218

[B87] Yoshinari Y. Kosakamoto H. Kamiyama T. Hoshino R. Matsuoka R. Kondo S. (2021). The sugar-responsive enteroendocrine neuropeptide F regulates lipid metabolism through glucagon-like and insulin-like hormones in *Drosophila melanogaster*. *Nat. Commun.* 12:4818. 10.1038/s41467-021-25146-w 34376687 PMC8355161

[B88] Zhu Z. Tsuchimoto M. Nagata S. (2022). CCHamide-2 signaling regulates food intake and metabolism in *Gryllus bimaculatus*. *Insects* 13:324. 10.3390/insects13040324 35447766 PMC9026500

