# Supplementary material for: Nutrient responding peptide hormone CCHamide-2 consolidates appetitive memory
Source: Front Behav Neurosci. 2022 Oct 19;16:986064. doi: 10.3389/fnbeh.2022.986064 (PMC9627028; doi:10.3389/fnbeh.2022.986064)
Supplement: Supplementary file 1 [file Table_1.DOCX]

Table S1. List of fly strains and crosses for behavioral experiments, and the statistical results

| Figure | Group code | Males | Females | *N* | *D'Agostino (Shapiro-Wilk) normality test*  *(P value)* | *Brown-Forsythe test (P value)* | *Effect size (f)* | *Achieved N*  *(Required N for power > .8)* | *Statistical test for group comparisons*  *(Adjusted P value)* |
| --- | --- | --- | --- | --- | --- | --- | --- | --- | --- |
| Figure 1A | *1* | *CS* | *CS* | *20* | *0.323* | *NA* | *0.352* | *76 (60)* | *0.0502 (1 vs. 2)*  *> 0.9999 (1 vs. 3)*  *0.0613 (1 vs. 4)*  *> 0.9999 (1 vs. 5)*  *> 0.9999 (1 vs. 6)*  *Dunn’s test* |
|  | *2* | *CCHa2 CR1* | *CS* | *8* | *0.2218* |  |  |  |  |
|  | *3* | *CCHa2 CR3* | *CS* | *8* | *0.8683* |  |  |  |  |
|  | *4* | *CCHa2 CR1* | *CCHa2 CR1* | *16* | *< 0.0001 **** |  |  |  |  |
|  | *5* | *CCHa2 CR3* | *CCHa2 CR3* | *16* | *0.065* |  |  |  |  |
|  | *6* | *CCHa2 CR1* | *CCHa2 CR3* | *8* | *0.1908* |  |  |  |  |
| Figure 1B | *1* | *CS* | *CS* | *15* | *0.3802* | *NA* | *0.908* | *88 (60)* | *0.9943 (1 vs. 2)*  *0.9505 (1 vs. 3)*  *< 0.0001 (1 vs. 4) ****  *< 0.0001 (1 vs. 5) ****  *0.049 (1 vs. 6) **  *Dunnett's test* |
|  | *2* | *CCHa2 CR1* | *CS* | *14* | *0.1114* |  |  |  |  |
|  | *3* | *CCHa2 CR3* | *CS* | *15* | *0.2503* |  |  |  |  |
|  | *4* | *CCHa2 CR1* | *CCHa2 CR1* | *16* | *0.7305* |  |  |  |  |
|  | *5* | *CCHa2 CR3* | *CCHa2 CR3* | *14* | *0.4596* |  |  |  |  |
|  | *6* | *CCHa2 CR1* | *CCHa2 CR3* | *14* | *0.9312* |  |  |  |  |
| Figure 2A | *1* | *CCHa2-GAL4* | *w* | *17* | 0.4039 | *0.6422* | *0.124* | *48 (42)* | *0.8615 (1 vs. 2)*  *0.9260 (2 vs. 3)*  *Sidak's test* |
|  | *2* | *CCHa2-GAL4* | *UAS-Shibire[ts1]* | *15* | 0.9959 |  |  |  |  |
|  | *3* | *w* | *UAS-Shibire[ts1]* | *16* | 0.3535 |  |  |  |  |
| Figure 2B | *1* | *CCHa2-GAL4* | *w* | *16* | *0.12* | *0.3919* | *0.477* | *47 (42)* | *0.0125 (1 vs. 2) **  *0.0328 (2 vs. 3) **  *Sidak's test* |
|  | *2* | *CCHa2-GAL4* | *UAS-Shibire[ts1]* | *16* | *0.6599* |  |  |  |  |
|  | *3* | *w* | *UAS-Shibire[ts1]* | *15* | *0.4329* |  |  |  |  |
| Figure 2C | *1* | *CCHa2-GAL4* | *w* | *20* | *0.2799* | *NA* | *0.149* | *58 (42)* | *> 0.9999 (1 vs. 2)*  *0.7323 (2 vs. 3)*  *Dunn’s test* |
|  | *2* | *CCHa2-GAL4* | *UAS-Shibire[ts1]* | *22* | *0.0373 ** |  |  |  |  |
|  | *3* | *w* | *UAS-Shibire[ts1]* | *16* | *0.8322* |  |  |  |  |
| Figure 3A | *1* | *CCHa2-GAL4* | *w* | *13* | *0.8805* | *0.2984* | *0.404* | *48 (42)* | *0.0358 (1 vs. 2) **  *0.0444 (2 vs. 3) **  *Sidak's test* |
|  | *2* | *CCHa2-GAL4* | *UAS-dTrpA1* | *18* | *0.8237* |  |  |  |  |
|  | *3* | *w* | *UAS-dTrpA1* | *17* | *0.6666* |  |  |  |  |
| Figure 3B | *1* | *CCHa2-GAL4* | *w* | *16* | 0.2696 | *0.1483* | *0.078* | *45 (42)* | *0.9591 (1 vs. 2)*  *0.9591 (2 vs. 3)*  *Sidak's test* |
|  | *2* | *CCHa2-GAL4* | *UAS-dTrpA1* | *14* | 0.0827 |  |  |  |  |
|  | *3* | *w* | *UAS-dTrpA1* | *15* | 0.1571 |  |  |  |  |
| Figure 6A | *1* | *R58E02-GAL4* | *w* | *10* | *0.203* | *0.2494* | *0.497* | *34 (42)* | *0.1807 (1 vs. 2)*  *0.0189 (2 vs. 3) **  *Holm-Sidak's test* |
|  | *2* | *R58E02-GAL4* | *UAS-CCHa2-R-RNAi* | *10* | *0.69* |  |  |  |  |
|  | *3* | *w* | *UAS-CCHa2-R-RNAi* | *14* | *0.2722* |  |  |  |  |
| Figure 6B | *1* | *R58E02-GAL4* | *w* | *15* | *0.8792* | *NA* | *0.477* | *46 (42)* | *0.0025 (1 vs. 2) ***  *0.0056 (2 vs. 3) ***  *Dunn’s test* |
|  | *2* | *R58E02-GAL4* | *UAS-CCHa2-R-RNAi* | *15* | *0.2683* |  |  |  |  |
|  | *3* | *w* | *UAS-CCHa2-R-RNAi* | *16* | *< 0.0001 **** |  |  |  |  |
| Figure 6C | *1* | *MB299B-GAL4* | *w* | *7* | *NA* | *0.4547* | *0.832* | *23 (42)* | *0.4247 (1 vs. 2)*  *0.0026 (2 vs. 3) ***  *Sidak’s test* |
|  | *2* | *MB299B-GAL4* | *UAS-CCHa2-R-RNAi* | *7* | *NA* |  |  |  |  |
|  | *3* | *W* | *UAS-CCHa2-R-RNAi* | *9* | *0.394* |  |  |  |  |
| Figure 6D | *1* | *MB299B-GAL4* | *w* | *14* | *0.9577* | *0.1934* | *0.574* | *40 (42)* | *0.0039 (1 vs. 2) ***  *0.0208 (2 vs. 3) **  *Holm-Sidak's test* |
|  | *2* | *MB299B-GAL4* | *UAS-CCHa2-R-RNAi* | *13* | *0.1675* |  |  |  |  |
|  | *3* | *w* | *UAS-CCHa2-R-RNAi* | *13* | *0.4375* |  |  |  |  |
