# Supplementary figures and images for: Nutrient responding peptide hormone CCHamide-2 consolidates appetitive memory
Source: Front Behav Neurosci. 2022 Oct 19;16:986064. doi: 10.3389/fnbeh.2022.986064 (PMC9627028; doi:10.3389/fnbeh.2022.986064)

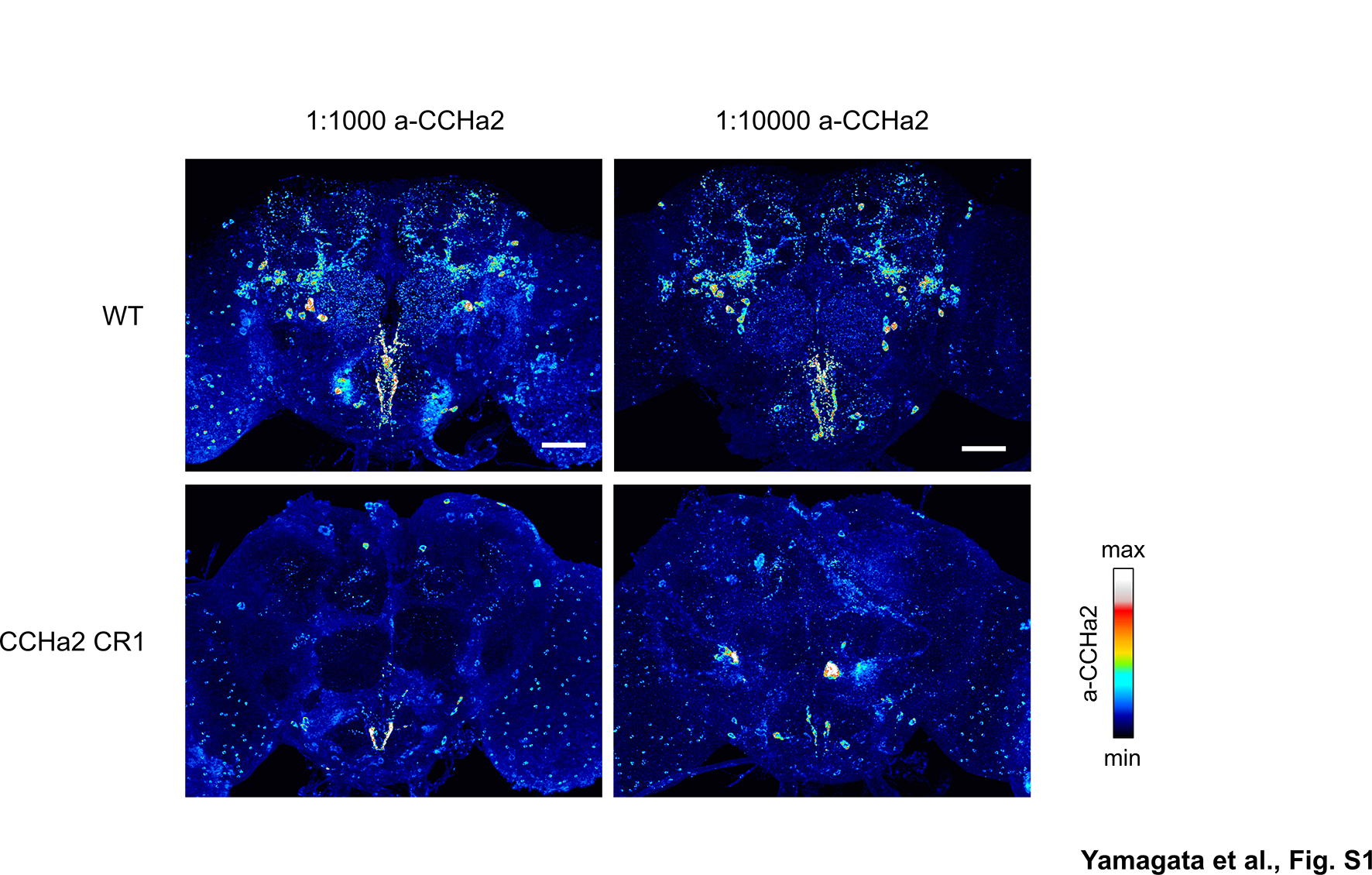

Supplement: Supplementary Figure 1 — Antibody staining of CCHa2 in the wild type (upper) and CCHa2 CR1 mutant (lower) brains. The a-CCHa2 signal was largely diminished in the mutant brain except for a small fraction of cells in the medial protocerebrum, the optic lobe, the tritocerebrum, and the gnathal ganglia, suggestive of reasonable specificity of the antibody. No apparent pattern difference was observed in the two staining conditions, though there was less background signal in the lower dilution. [file Image_1.TIF]
